# Komplette Lymphknotendissektion versus selektive Lymphknotenexstirpation bei Melanompatienten mit nodaler Makrometastasierung und adjuvanter Systemtherapie

**DOI:** 10.1111/ddg.15967_g

**Published:** 2026-08-04

**Authors:** Kristine E. Mayer, Jessica C. Hassel, Jannik Sambale, Michael Erdmann, Mihaela‐Anca Sindrilaru, Julia Oberschmied, Alexander Thiem, Dirk Tomsitz, Jana Knuever, Max Schlaak, Carola Berking, Christian Posch, Tilo Biedermann, Oana‐Diana Persa

**Affiliations:** ^1^ Abteilung für Dermatologie und Allergologie Medizinische Fakultät Technische Universität München (TUM) Bayerisches Zentrum für Krebsforschung (BZKF) München Deutschland; ^2^ Abteilung für Dermatologie Medizinische Fakultät Heidelberg Nationales Centrum für Tumorerkrankungen (NCT) NCT Heidelberg eine Partnerschaft zwischen dem DKFZ und dem Universitätsklinikum Heidelberg Heidelberg Deutschland; ^3^ Abteilung für Dermatologie Universitätsklinikum Erlangen Comprehensive Cancer Center Erlangen – EMN Friedrich‐Alexander‐Universität Erlangen‐Nürnberg (FAU) CCC‐Allianz WERA Bayerisches Zentrum für Krebsforschung (BZKF) Erlangen Deutschland; ^4^ Abteilung für Dermatologie und Allergologie Universitätsklinikum Ulm Ulm Deutschland; ^5^ Klinik für Dermatologie Venerologie und Allergologie Charité – Universitätsmedizin Berlin Körperschaft des öffentlichen Rechts Mitglied der Freien Universität Berlin und der Humboldt‐Universität zu Berlin Berlin Deutschland; ^6^ Abteilung für Dermatologie und Venerologie Universitätsmedizin Rostock Rostock Deutschland; ^7^ Abteilung für Dermatologie und Allergologie LMU Klinikum Ludwig‐Maximilians‐Universität München München Deutschland; ^8^ Abteilung für Dermatologie und Venerologie Universitätsklinikum Köln Köln Deutschland; ^9^ Abteilung für Dermatologie und Allergologie Zentrum für Hauterkrankungen Universitätsklinikum Bonn Bonn Deutschland; ^10^ Abteilung für Medizin Sigmund‐Freud‐Privatuniversität Wien Österreich; ^11^ Abteilung für Dermatologie Klinik Hietzing Wiener Gesundheitsverbund Wien Österreich

**Keywords:** Adjuvante Therapie, Lymphknotenexstirpation, Lymphknotendissektion, Lymphknotenmetastasierung, Makrometastasierung, Melanom, Adjuvant therapy, lymph node dissection, lymph node extirpation, lymph node metastasis, macrometastasis, melanoma

## Abstract

**Hintergrund und Zielsetzung:**

Die komplette Lymphknotendissektion (CLND) gilt als Standardbehandlung bei Melanompatienten mit regionalen Lymphknotenmakrometastasen. Es fehlt jedoch an Evidenz bezüglich des chirurgischen Vorgehens im Kontext adjuvanter Systemtherapien.

**Patienten und Methodik:**

Diese retrospektive multizentrische Studie schloss Melanompatienten im Stadium IIIB–D mit nodaler Makrometastasierung ein, die vor einer adjuvanten Therapie eine CLND oder selektive Lymphknotenexstirpation (LNE) erhalten hatten. CLND und LNE wurden bezüglich des rezidivfreien Überlebens (RFS), des Lymphknotenmetastasen‐freien Überlebens (NFS) und des Gesamtüberlebens (OS) verglichen.

**Ergebnisse:**

320 Patienten wurden eingeschlossen (medianes Alter 62 Jahre; 55,5 % Männer). Die Patienten erhielten entweder eine PD‐1‐Monotherapie (77,8 %), eine zielgerichtete Therapie (21,3 %) oder beides sequenziell (0,9 %) sowie eine adjuvante Strahlentherapie in 40,9 % der Fälle. RFS und OS unterschieden sich nicht signifikant nach CLND oder LNE, während das NFS nach CLND signifikant verlängert war (HR 0,3917; p = 0,005). Unter Berücksichtigung von Risikofaktoren mittels multivariater Cox‐Regression zeigte sich ein verlängertes RFS für CLND gegenüber LNE (HR 0,676; p = 0,04), aber kein Vorteil im OS. Die Komplikationsrate war in der CLND‐Gruppe signifikant erhöht.

**Schlussfolgerungen:**

Die CLND zeigte keinen Gesamtüberlebensvorteil im Vergleich zur LNE, obwohl die lokale Kontrolle verbessert wurde. Die Durchführung einer CLND kann im Kontext adjuvanter Therapien empfohlen werden, jedoch sollte das erhöhte Risiko für Komplikationen bedacht werden.

## EINLEITUNG

Melanompatienten mit dicken Primärtumoren oder regionalen Metastasen haben nach chirurgischer Resektion ein hohes Rezidivrisiko, das zwischen 35 % im Stadium IIB und 79 % im Stadium IIID liegt.^1^ Daher wird diesen Patienten in der Regel eine adjuvante Therapie angeboten, entweder mit PD‐1 blockierenden Immuncheckpoint‐Inhibitoren (ICI)^2^, ^3^, ^4^ oder, im Falle einer aktivierenden BRAFV600‐Mutation und einer Erkrankung im Stadium III, einer Kombination aus BRAF‐ und MEK‐Inhibitoren.^5^, ^6^ Beide adjuvante Therapiekonzepte weisen eine ähnliche Wirksamkeit bezüglich der Verringerung des relativen Rezidivrisikos von 0,61 für anti‐PD‐1,^2^ und von 0,51 für die zielgerichtete Therapie auf.^5^ Patienten mit einer Erkrankung im Stadium III, die in die klinischen Studien zu den adjuvanten Therapien eingeschlossen wurden, hatten zuvor eine vollständige Lymphknotendissektion (CLND) als Einschlusskriterium gemäß Protokoll erhalten. Aus diesem Grund ist bis heute nicht bekannt, ob Patienten unter adjuvanter Systemtherapie von einer CLND profitieren oder ob der klinische Verlauf vergleichbar wäre, wenn ausschließlich die Lymphknotenmetastasen (LNE) entfernt würden. Zuletzt konnten mittels neoadjuvanter Behandlungskonzepte hervorragende Raten an pathologischem Ansprechen und ereignisfreiem Überleben nach neoadjuvantem Einsatz der Kombination von Ipilimumab und Nivolumab oder der Monotherapie mit Pembrolizumab erzielt werden.^7^, ^8^, ^9^ Dadurch ist die Rolle der CLND mit anschließender adjuvanter Behandlung bei einer Subgruppe von Patienten mit Melanom im Stadium III zu hinterfragen. Während die meisten Patienten von einer neoadjuvanten Behandlung profitieren, gibt es jedoch auch Patienten, deren Erkrankung unter der Behandlung fortschreitet. Dies deutet darauf hin, dass die CLND auch in Zukunft noch eine Rolle bei der Behandlung von Patienten im Stadium III spielen wird.

Bei Patienten mit Mikrometastasierung in der Sentinel‐Lymphknoten‐Biopsie (SLNB) wird die CLND nicht mehr empfohlen. Randomisierte Studien konnten zeigen, dass die CLND weder das Risiko für ein Rezidiv noch für eine Fernmetastasierung reduziert und keinen signifikanten Einfluss auf das Gesamtüberleben (OS) hat.^10^, ^11^, ^12^ Dies konnte durch retrospektive Analysen bestätigt werden.^13^, ^14^, ^15^, ^16^, ^17^ Auch bei Patienten mit mehreren positiven Sentinel‐Lymphknoten (SLN) konnte die CLND das melanomspezifische Überleben und das rezidivfreie Überleben (RFS) nicht signifikant verbessern.^17^

Der therapeutische Nutzen des chirurgischen Vorgehens bei Patienten mit Lymphknoten‐Makrometastasen ist bis heute unklar. Vor Einführung der adjuvanten Behandlung mit PD‐1‐ und BRAF/MEK‐Inhibitoren wurde aufgrund fehlender therapeutischer Alternativen häufig eine CLND durchgeführt.^18^, ^19^, ^20^ Aus damaligen Studien zur CLND ist bekannt, dass unter anderem die Anzahl der betroffenen Lymphknoten und die Dicke des Primärtumors das RFS und das OS negativ beeinflussen.^18^, ^21^ Andererseits scheint eine größere Anzahl an exzidierten Lymphknoten bei Patienten im Stadium III mit einer besseren Prognose assoziiert zu sein.^22^, ^23^ In der Kohorte von Rossi und Kollegen, die 2507 Patienten im Stadium III umfasste, lag die mediane Anzahl exzidierter Lymphknoten bei 15, abhängig von der anatomischen Lage.^22^ Eine multivariate Subgruppenanalyse ergab, dass Patienten mit Mikrometastasen, nicht aber Patienten mit Makrometastasen, von einer größeren Anzahl entfernter Lymphknoten profitierten.^22^ Darüber hinaus wurde nur in der Subgruppe von Patienten mit 2–3 positiven Lymphknoten ein prognostischer Vorteil einer größeren Anzahl entfernter Lymphknoten beobachtet.^22^ Im Kontext effektiver adjuvanter Systemtherapien bleibt daher zu beantworten, ob Patienten mit Lymphknoten‐Makrometastasen von einer regionalen CLND profitieren oder ob die Durchführung einer CLND im Vergleich zur LNE den klinischen Verlauf nicht wesentlich beeinflusst.

Neben möglichen prognostischen Vorteilen ist die CLND mit einer höheren Morbidität assoziiert.^24^ So können bei Lymphknotenoperationen langfristige Komplikationen wie Lymphödeme oder Lähmungen aufgrund von Nervenschäden auftreten. Frühere Studien haben gezeigt, dass eine größere Anzahl exzidierter Lymphknoten mit einer größeren Anzahl an postoperativen Komplikationen korreliert.^25^ Deswegen sollte eine CLND bei fehlendem Überlebensvorteil vermieden werden, was etwa beim Vergleich der ilioinguinalen mit der inguinalen Lymphadenektomie gezeigt wurde.^26^, ^27^

In dieser retrospektiven multizentrischen Studie wurde das Überleben bei Patienten mit Lymphknoten‐Makrometastasen im Zeitalter moderner adjuvanter Therapien nach CLND beziehungsweise LNE untersucht. Ziel der Studie war es, die klinische Entscheidungsfindung bezüglich des chirurgischen Vorgehens bei Makrometastasen zu erleichtern, indem evidenzbasierte Vor‐ und/oder Nachteile von CLND beziehungsweise LNE identifiziert werden.

## PATIENTEN UND METHODIK

### Studiendesign

In diese retrospektive Studie wurden Melanompatienten in den Stadien IIIB–IIID nach AJCCv8 eingeschlossen, die entweder eine CLND oder eine LNE zwischen 2015 und 2024 an einer der acht teilnehmenden Universitätskliniken mit Spezialisierung auf Dermatoonkologie in Deutschland erhalten hatten. Die Studie wurde von der Ethikkommission der Technischen Universität München genehmigt. Zu den Einschlusskriterien zählten die Diagnose eines histologisch gesicherten Melanoms, der klinische Verdacht auf eine Lymphknoten‐Makrometastase, das Fehlen von Fernmetastasen zum Zeitpunkt des chirurgischen Eingriffs, die Durchführung einer CLND oder LNE, die histologische Bestätigung mindestens einer Lymphknotenmetastase und eine adjuvante systemische Behandlung mit einem PD‐1‐Inhibitor, einer BRAF/MEK‐Inhibition oder beider Therapien aufeinander folgend. Lymphknotenexstirpation war definiert als chirurgische Resektion von ausschließlich prä‐ und intraoperativ klinisch verdächtigen Lymphknoten, CLND umfasste die systematische inguinale, axilläre oder zervikale Lymphadenektomie.

Neben Patienten‐ und Tumorcharakteristika sowie Behandlungsschemata wurden folgende Parameter erhoben: Anzahl der resezierten Lymphknoten, Anzahl der histologisch bestätigten Lymphknotenmetastasen, Durchmesser der größten Lymphknotenmetastase, Vorhandensein einer extrakapsulären Ausbreitung, Durchführung einer adjuvanten Strahlentherapie, Durchführung und Dauer einer adjuvanten Systemtherapie, mit den chirurgischen Eingriffen verbundene Nebenwirkungen, Auftreten eines Rezidivs oder eines Todesfalls und entsprechende Zeit seit dem Eingriff, Art von Progress sowie Länge des Nachbeobachtungszeitraums. Primäres Ziel der Studie war es, die Auswirkungen der CLND oder der selektiven LNE auf RFS, Lymphknotenmetastasen‐freien Überlebens (NFS) und OS bei Patienten mit Lymphknoten‐Makrometastasen zu untersuchen. Das RFS war definiert als der Zeitraum von der Intervention (CLND/LNE) bis zum Auftreten eines Rezidivs, während NFS als der Zeitraum von der Intervention bis zur Feststellung einer lokoregionären Lymphknotenmetastase definiert war. Das OS war definiert als der Zeitraum von der Intervention bis zum Tod. Patienten, bei denen kein Ereignis auftrat, wurden beim letzten Patientenkontakt zensiert.

### Statistik

GraphPad Prism v10.4.0 und IBM SPSS Statistics v29.0.2 wurden zur Darstellung der Ergebnisse und zur Berechnung der statistischen Tests eingesetzt. Um das Überleben zwischen den Gruppen zu vergleichen, wurden zweiseitige Log‐Rank‐Tests von Kaplan‐Meier‐Kurven durchgeführt und das mediane RFS, NFS und OS bestimmt. Der mediane Nachbeobachtungszeitraum wurde anhand des Zeitraums zwischen der Intervention (CLND/LNE) und dem Tod oder dem letzten Patientenkontakt errechnet.

Die unbereinigten Risikoschätzungen verschiedener Parameter, einschließlich der Interventionen, für RFS und OS wurden durch eine univariate Cox‐Proportional‐Hazards‐Regressionsanalyse bestimmt. Zu den Parametern gehörten Geschlecht, Alter, Tumordicke, Ulzeration, BRAF‐Mutation, Laktatdehydrogenase (LDH)‐Spiegel (erhöht vs. nicht erhöht) zu Beginn der adjuvanten Therapie, Anzahl der resezierten Lymphknoten, mehr als drei metastatische Lymphknoten (ja vs. nein), Durchmesser der größten Lymphknotenmetastase, extrakapsuläre Ausbreitung (ja vs. nein), chirurgischer Eingriff (CNLD vs. LNE), adjuvante Strahlentherapie (ja vs. nein), adjuvante Systemtherapie (BRAF/MEK‐Hemmung vs. PD‐1‐Inhibitor) und die Dauer der adjuvanten Systemtherapie in Monaten.

Das rezidivfreie Überleben und das Gesamtüberleben wurden mithilfe eines zeitabhängigen multivariaten Cox‐Proportional‐Hazards‐Modells bewertet, das nach mehr als drei metastasierten Lymphknoten, dem Alter und der Dauer der adjuvanten Systemtherapie stratifiziert war und den Eingriff (CLND vs. LNE) als Faktor einschloss. Die so errechneten Hazard Ratios (HR) mit zugehörigen 95 %‐Konfidenzintervallen (95 %‐KI) sowie die p‐Werte waren für das Vorhandensein aller Faktoren angepasst. Für alle Analysen wurde die Schwelle für die statistische Signifikanz auf p < 0,05 festgelegt.

## ERGEBNISSE

### Patientencharakteristika

**ABBILDUNG 1:**
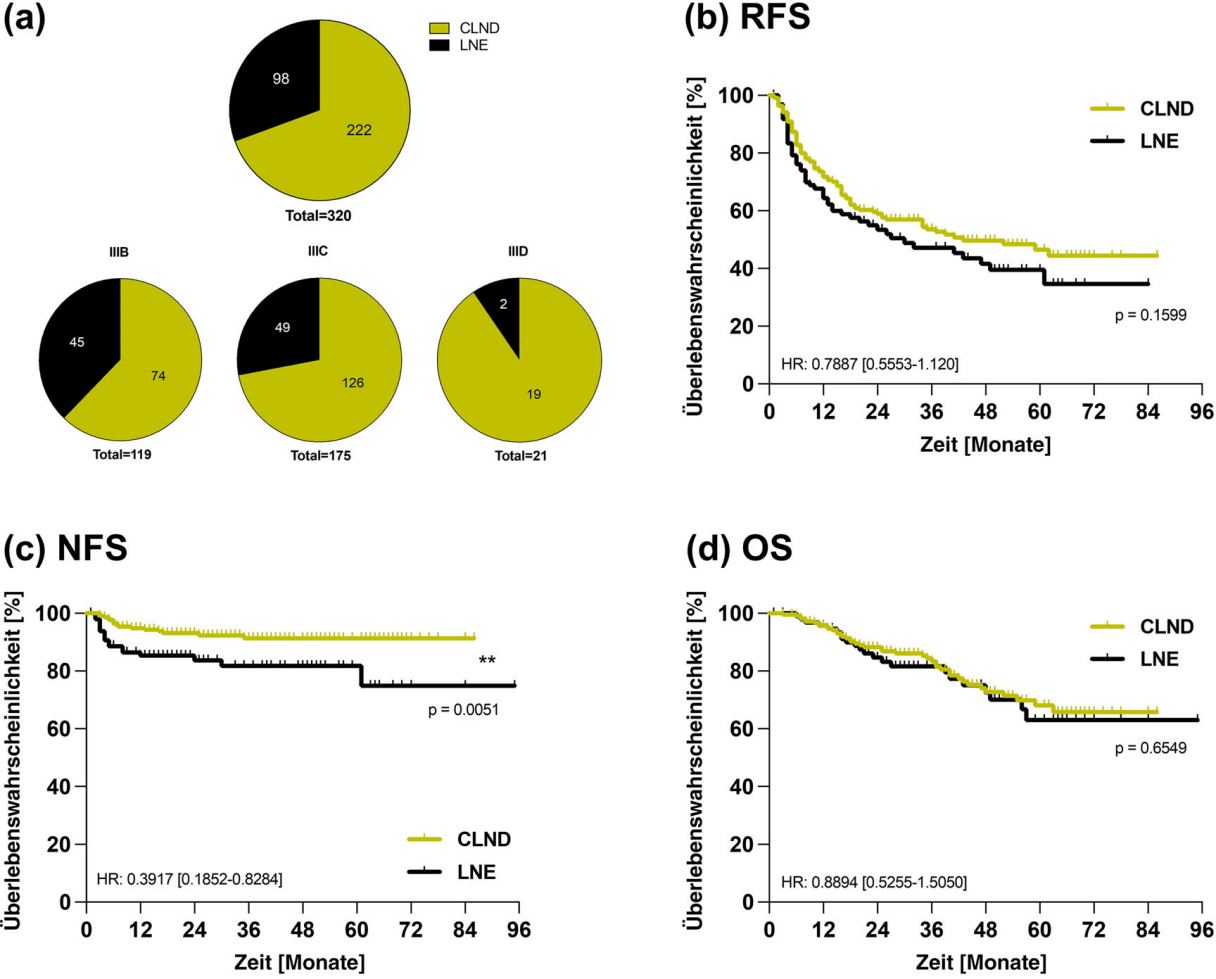
Verteilung der Krankheitsstadien und des Überlebens für die gesamte Studienkohorte nach CLND und LNE. (a) Verteilung der Patienten, die sich einer CLND im Vergleich zu einer LNE unterzogen, für die gesamte Studienkohorte und nach AJCCv8‐Stadien IIIB, IIIC und IIID. (b) Kaplan‐Meier‐Kurven des rezidivfreien Überlebens (RFS), (c) des nodalrezidivfreien Überlebens (NFS) und (d) des Gesamtüberlebens (OS) für die gesamte Studienkohorte je nach Eingriff (CLND vs. LNE). Die Hazard Ratios (HR) einschließlich ihres 95 %‐Konfidenzintervalls [95 %‐KI] sind in der unteren linken Ecke jeder Grafik angegeben.

In die Analyse wurden insgesamt 320 Patienten mit Lymphknoten‐Makrometastasen (AJCCv8‐Stadium IIIB–IIID) eingeschlossen, die eine CLND oder LNE erhalten hatten. Davon erhielten 222 (69,4 %) Patienten eine CLND, während 98 (30,6 %) Patienten eine LNE erhielten. Detaillierte Informationen zu den Patientencharakteristika finden sich in Tabelle 1. Die beiden Gruppen waren sehr heterogen. Die Anzahl der histologisch gesicherten Lymphknotenmetastasen war in der CLND‐Gruppe größer. Entsprechend war die Verteilung Krankheitsstadien nach AJCCv8 zwischen den beiden Gruppen ebenfalls unterschiedlich. Bei Patienten, bei denen später eine Erkrankung im Stadium IIIC und IIID diagnostiziert wurde, wurde häufiger einer CLND durchgeführt (Abbildung 1a). Andererseits war in der LNE‐Gruppe der Anteil der Patienten im Stadium IIIB höher und nur zwei Patienten wurden als Stadium IIID eingestuft (Tabelle 1). Die mediane Nachbeobachtungszeit betrug 34 Monate in der CLND‐Gruppe und 37 Monate in der LNE‐Gruppe.

**TABELLE 1:** Patientenmerkmale der gesamten Studienkohorte. Charakteristika der Studienkohorte (n = 320): vollständige Lymphknotendissektion (CLND) oder selektive Lymphknotenexstirpation (LNE) verdächtiger Lymphknoten. Der LDH‐Serumspiegel ist in den Kategorien normal und erhöht (über der oberen Grenze des Normalwerts) angegeben.

	CLND (n = 222; 69,4 %)	LNE (n = 98; 30,6 %)
***Alter (Jahre)***		
Median (Range)	62 (24–88)	64 (28–87)
***Geschlecht***		
Weiblich	90 (40,5 %)	53 (54,1 %)
Männlich	132 (59,5 %)	45 (45,9 %)
***BRAF‐Status***		
V600‐Wildtyp	107 (48,2 %)	59 (60,2 %)
V600‐Mutation	103 (46,4 %)	37 (37,8 %)
Unbekannt	12 (5,4 %)	2 (2,0 %)
***Stadium (AJCCv8)***		
IIIB	74 (33,3 %)	45 (45,9 %)
IIIC	126 (56,8 %)	49 (50,0 %)
IIID	19 (8,6 %)	2 (2,0 %)
Unbekannt	3 (1,4 %)	2 (2,0 %)
***Tumordicke (mm)***		
Mittelwert (Range)	4,23 (0,2–17)	3,52 (0,3–11)
***Ulzeration des Primärtumors***		
Ja	78 (35,1 %)	34 (34,7 %)
Nein	109 (49,1 %)	42 (42,9 %)
Unbekannt/nicht zutreffend	35 (15,8 %)	22 (22,4 %)
***Anzahl entfernter Lymphknoten***		
Mittelwert	14,83	2,55
Standardabweichung	9,28	4,12
***Anzahl histologisch bestätigter Lymphknotenmetastasen***		
Mittelwert	3,86	1,35
Standardabweichung	3,87	0,91
***Durchmesser der größten Lymphknotenmetastase (cm)***		
Mittelwert	2,85	2,45
Standardabweichung	2,30	1,67
***Extrakapsuläre Ausbreitung***		
Ja	80 (36,0 %)	33 (33,7 %)
Nein	125 (56,3 %)	54 (55,1 %)
Unbekannt	17 (7,7 %)	11 (11,2 %)
***LDH im Serum***		
Normal (≤ obere Grenze des Normwerts)	168 (75,7 %)	74 (75,5 %)
Erhöht (> obere Grenze des Normwerts)	33 (14,9 %)	15 (15,3 %)
Unbekannt	21 (9,5 %)	9 (9,2 %)
***Adjuvante Systemtherapie***		
PD‐1 Inhibitor	167 (75,2 %)	82 (83,7 %)
BRAF/MEK Inhibitor	53 (23,9 %)	15 (15,3 %)
Beides nacheinander	2 (0,9 %)	1 (1,0 %)
***Dauer der adjuvanten Systemtherapie (Monate)***		
Mittelwert	8,59	8,28
Standardabweichung	4,40	4,55

*Abk*.: AJCCv8, 8. Auflage des American Joint Committee on Cancer; BRAF, B‐Raf proto‐oncogene serine/threonine kinase; CLND, vollständige Lymphknotendissektion; LNE, Lymphknotenexstirpation; LDH, Laktatdehydrogenase; MEK, Mitogen‐activated protein kinase kinase; PD‐1, Programmed cell death protein 1

### Die CLND ist mit einem signifikant verlängertem NFS, nicht aber RFS und OS in der Gesamtkohorte assoziiert

Trotz erheblicher Unterschiede in der Verteilung der Krankheitsstadien zwischen der CLND‐ und der LNE‐Gruppe ergaben zweiseitige Log‐Rank‐Tests keine statistisch signifikanten Unterschiede für RFS (HR 0,7887 [0,5553–1,120], p = 0,16) und OS (HR 0,8894 [0,5255–1,5050], p = 0,65) zwischen den beiden Gruppen (Abbildung 1b, d). Das NFS war jedoch in der CLND‐Gruppe im Vergleich zur LNE‐Gruppe signifikant verlängert (HR 0,3917 [0,1852–0,8284], p = 0,005) (Figure 1c). Das mediane RFS betrug 43 Monate in der CLND‐Gruppe und 30 Monate in der LNE‐Gruppe (Tabelle S1 im Online‐Supplement). Mediane NFS und OS wurden in beiden Gruppen nicht erreicht (Online‐Tabelle S1 im Online‐Supplement).

Die Analyse des Typs von Progress (Mehrfachnennungen möglich) zeigte, dass durch die CLND nur die lokoregionäre lymphatische Metastasierung besser kontrolliert war (16,3 % Rezidive mit Lymphknotenmetastasen in der CLND‐Gruppe gegenüber 33,3 % in der LNE‐Gruppe; p = 0,006). Dies denkt sich mit dem beobachteten verbesserten NFS (Figure 1c). Die Raten an Patienten mit einem Progress in Form von Fernmetastasierung waren jedoch zwischen den beiden Gruppen vergleichbar (75,5 % beziehungsweise 70,6 %; p = 0,55). Auch die Raten an In‐Transit‐Metastasierung oder Lokalrezidiven im Bereich des Primärtumors waren vergleichbar, wenn auch mit geringer Inzidenz (11,2 % vs. 13,7 %; p = 0,43) (Tabelle S1 im Online‐Supplement). Die Zweitlinientherapie bei einem Rezidiv nach einer LNE bestand überwiegend aus Systemtherapien (96 %), während eine Zweitlinien‐CLND nur bei 17 % der Patienten durchgeführt wurde.

### Die CLND ist unter Berücksichtigung relevanter Kovariaten mit verlängertem RFS assoziiert

Mit Hilfe einer univariaten Cox‐Proportional‐Hazard‐Regressionsanalyse wurden die Auswirkungen verschiedener klinischer Merkmale und Behandlungsfaktoren auf das RFS untersucht (Tabelle 2). Das Alter, das Geschlecht, der BRAF‐Mutationsstatus, die LDH‐Werte, die Anzahl der entfernten Lymphknoten, der Durchmesser der größten Lymphknotenmetastase und die Art der adjuvanten Systemtherapie (BRAF/MEK‐Inhibition oder PD‐1‐Inhibition) zeigten keinen signifikanten Einfluss auf das RFS. Das Vorhandensein einer extrakapsulären Ausbreitung, mehr als drei metastatische Lymphknoten, eine adjuvante Strahlentherapie (diese wird bei Vorliegen der oben genannten Risikofaktoren empfohlen) und eine kürzere Dauer der Systemtherapie waren jedoch mit einer signifikant kürzeren RFS assoziiert. Um die großen klinischen Unterschiede zwischen der CLND‐ und der LNE‐Gruppe zu berücksichtigen, wurde ein multivariates Regressionsmodell eingeführt, für welches die durch die univariate Cox‐Proportional‐Hazards‐Regressionsanalyse ermittelten, relevanten Kovariaten herangezogen wurden. Die CLND zeigte im Vergleich zur LNE ein geringeres Rezidivrisiko (HR 0,676 [0,468–0,976], p = 0,04) (Tabelle 3). Weitere unabhängige Risikofaktoren für einen Progress waren mehr als drei metastatische Lymphknoten (HR 2,210 [1,492–3,273], p < 0,001) und die Dauer der adjuvanten Systemtherapie (HR 0,834 [0,805–0,865], p < 0,001).

**TABELLE 2:** Univariate Cox‐Proportional‐Hazard‐Regressionsmodelle für das rezidivfreie Überleben (RFS). p‐Werte, die Signifikanz anzeigen, sind kursiv dargestellt.

	Univariate Analyse: HR (95 %‐KI)	p‐Wert
**Allgemeine Charakteristika**		
Alter (Jahre)	1,002 (0,991–1,014)	0,68
Geschlecht (weiblich vs. männlich)	1,077 (0,780–1,485)	0,65
Tumordicke (mm)	1,000 (0,976–1,025)	>0,99
Ulzeration (ja vs. nein)	1,273 (0,898–1,806)	0,18
BRAF‐Mutation (ja vs. nein)	0,878 (0,633–1,219)	0,44
LDH vor Systemtherapie (erhöht vs. normal)	1,012 (0,633–1,617)	0,96
**Lymphatische Metastasierung**		
Anzahl resezierter Lymphknoten	0,995 (0,978–1,012)	0,54
Mehr als 3 Lymphknotenmetastasen (ja vs. nein)	1,727 (1,209–2,467)	*0,003*
Durchmesser der größten Lymphknotenmetastase (cm)	1,041 (0,958–1,131)	0,34
Extrakapsuläre Ausbreitung (ja vs. nein)	1,708 (1,216–2,400)	*0,002*
**Therapie**		
Intervention (CLND vs. LNE)	0,793 (0,566–1,110)	0,18
Adjuvante Strahlentherapie (ja vs. nein)	1,470 (1,066–2,028)	*0,02*
Adjuvante Systemtherapie (BRAF/MEK vs. PD‐1 Inhibitor)	0,723 (0,482–1,086)	0,12
Dauer der adjuvanten Systemtherapie (Monate)	0,836 (0,807–0,867)	*< 0,001*

*Abk*.: BRAF, B‐Raf proto‐oncogene serine/threonine kinase; CLND, vollständige Lymphknotendissektion; HR, Hazard Ratio; KI, Konfidenzintervall; LDH, Laktatdehydrogenase; LNE, Lymphknotenexstirpation; MEK, Mitogen‐activated protein kinase kinase; PD‐1, Programmed cell death protein 1

**TABELLE 3:** Multivariates Cox‐Proportional‐Hazard‐Regressionsmodell für das rezidivfreie Überleben (RFS). p‐Werte, die Signifikanz anzeigen, sind kursiv gedruckt.

	Multivariate Analyse: HR (95 %‐KI)	p‐Wert
Mehr als 3 Lymphknotenmetastasen (ja vs. nein)	2,210 (1,492–3,273)	*< 0,001*
Dauer der adjuvanten Systemtherapie (Monate)	0,834 (0,805–0,865)	*< 0,001*
Intervention (CLND vs. LNE)	0,676 (0,468–0,976)	*0,04*

*Abk*.: CLND, vollständige Lymphknotendissektion; HR, Hazard Ratio; KI, Konfidenzintervall; LNE, Lymphknotenexstirpation

### Die Dauer der adjuvanten Systemtherapie, aber nicht die Art der chirurgischen Intervention beeinflusst das OS bei Melanompatienten mit nodaler Makrometastasierung

Anschließend wurden verschiedene Faktoren, die das OS beeinflussen, mittels univariater Cox‐Proportional‐Hazards‐Regression identifiziert. Ein höheres Alter, der Nachweis einer extrakapsulären Ausbreitung der Lymphknotenmetastasen, eine adjuvante Strahlentherapie und eine kürzere Dauer der adjuvanten systemischen Therapie waren mit einem schlechteren OS assoziiert (Tabelle 4). Die multivariate Cox‐Regression ergab jedoch nur für die Dauer der adjuvanten systemischen Therapie einen signifikanten Einfluss auf das OS. Es wurde kein Vorteil der CLND im Vergleich zur LNE in Bezug auf das OS festgestellt (Tabelle 5).

**TABELLE 4:** Univariate Cox‐Proportional‐Hazard‐Regressionsmodelle für das Gesamtüberleben (OS). p‐Werte, die Signifikanz anzeigen, sind kursiv gedruckt.

	Univariate Analyse: HR (95 %‐KI)	p‐Wert
***Allgemeine Charakteristika***		
Alter (Jahre)	1,021 (1,002–1,040)	*0,03*
Geschlecht (weiblich vs. männlich)	1,023 (0,622–1,680)	0,93
Tumordicke (mm)	0,998 (0,963–1,034)	0,90
Ulzeration (ja vs. nein)	1,313 (0,776–2,221)	0,31
BRAF‐Mutation (ja vs. nein)	0,609 (0,361–1,028)	0,06
LDH vor Systemtherapie (erhöht vs. normal)	1,139 (0,572–2,268)	0,71
***Lymphatische Metastasierung***		
Anzahl resezierter Lymphknoten	0,995 (0,970–1,021)	0,71
Mehr als 3 Lymphknotenmetastasen (ja vs. nein)	1,538 (0,891–2,653)	0,12
Durchmesser der größten Lymphknotenmetastase (cm)	1,045 (0,918–1,191)	0,51
Extrakapsuläre Ausbreitung (ja vs. nein)	1,800 (1,073–3,020)	*0,03*
***Therapie***		
Intervention (CLND vs. LNE)	0,876 (0,523–1,468)	0,62
Adjuvante Strahlentherapie (ja vs. nein)	1,657 (1,014–2,710)	*0,04*
Adjuvante Systemtherapie (BRAF/MEK vs. PD‐1 Inhibitor)	0,500 (0,247–1,013)	0,054
Dauer der adjuvanten Systemtherapie (Monate)	0,845 (0,801–0,892)	*< 0,001*

**TABELLE 5:** Multivariates Cox‐Proportional‐Hazard‐Regressionsmodell für das Gesamtüberleben (OS). p‐Werte, die Signifikanz anzeigen, sind kursiv gedruckt.

	Multivariate Analyse: HR (95 %‐KI)	p‐Wert
Alter (Jahre)	1,016 (0,997–1,036)	0,09
Dauer der adjuvanten Systemtherapie (Monate)	0,847 (0,802–0,895)	*< 0,001*
Intervention (CLND vs. LNE)	1,111 (0,659–1,871)	0,69

### Die CLND ist mit mehr Komplikationen assoziiert

**ABBILDUNG 2:**
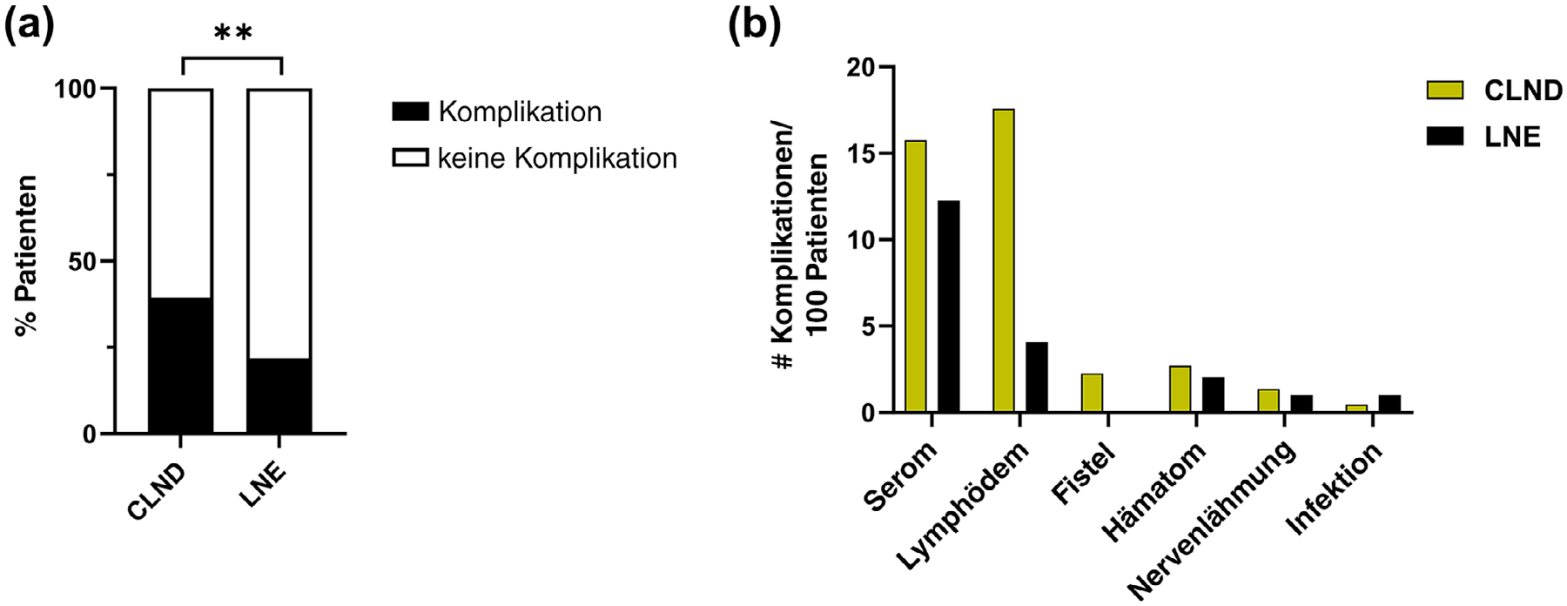
Postoperative Komplikationen nach CLND und LNE. (a) Anteil der Patienten, bei denen mindestens eine postoperative Komplikation nach CLND oder LNE auftrat. (b) Anzahl der Komplikationen pro 100 Patienten, aufgeschlüsselt nach Art der Komplikation, dargestellt für CLND und LNE.

Nachdem für die CLND‐Gruppe ein Vorteil in Bezug auf das NFS und RFS, nicht aber in Bezug auf das OS festgestellt wurde, sollte das Risiko für Komplikationen im Kontext beider Interventionen untersucht werden. Dazu wurde zunächst die Anzahl der Patienten, die mindestens eine postoperative Komplikation erlitten, bestimmt. Bei den Patienten, die eine CLND erhalten hatten, traten zu einem signifikant größeren Anteil Komplikationen auf als in der LNE‐Gruppe (Abbildung 2a). Die häufigsten Komplikationen waren Serome und Lymphödeme (Abbildung 2b, Tabelle S2 im Online‐Supplement). Während Serome auch in der LNE‐Gruppe eine häufige Komplikation darstellten, traten Lymphödeme vorwiegend nach CLND auf. Bei beiden Interventionen variierte die Komplikationsrate in Abhängigkeit von der Lokalisation des Eingriffs (Tabelle S2 im Online‐Supplement). Nach inguinalen Eingriffen wurde sowohl für die CLND‐Gruppe als auch für die LNE‐Gruppe eine signifikant höhere Komplikationsrate beobachtet als nach zervikalen Eingriffen (Chi‐Quadrat‐Test: p = 0,02 beziehungsweise p = 0,049). Trotz der Unterschiede in der Häufigkeit von Komplikationen zwischen den verschiedenen Lokalisationen waren die Ursachen für Komplikationen innerhalb der einzelnen Interventionsgruppen ähnlich verteilt (Tabelle S2 im Online‐Supplement). Insgesamt wurden Fistelbildung, Hämatome, Nervenparesen und Infektionen jeweils bei weniger als 5 % der Patienten beobachtet (Abbildung 2b).

## DISKUSSION

Dies ist nach unserem Kenntnisstand die erste Real‐World‐Studie, die den Überlebensvorteil von CLND gegenüber LNE bei Melanompatienten mit Lymphknoten‐Makrometastasen unter adjuvanter Systemtherapie untersucht. In dieser retrospektiven Studie wurde bei Patienten mit AJCCv8‐Stadien IIIC und IIID eine CLND bevorzugt, während bei Patienten mit Stadium IIIB häufiger eine LNE durchgeführt wurde. Das rezidivfreie Überleben und das Gesamtüberleben waren zwischen den beiden Gruppen vergleichbar, während die CLND mit einem verlängerten NFS assoziiert war. Eine multivariate Cox‐Regressionsanalyse, bei der die beiden Kovariaten mehr als drei betroffene Lymphknoten und die Dauer der adjuvanten Systemtherapie berücksichtigt wurden, zeigte jedoch eine signifikante Verlängerung des RFS nach CLND im Vergleich zur LNE. Die CLND hatte jedoch keinen signifikanten Einfluss auf das OS. Außerdem traten bei Patienten, die eine CLND erhalten hatten, häufiger Komplikationen auf, insbesondere chronische Lymphödeme.

Diese retrospektive Analyse ergab kein Vorteil für das Gesamtüberleben durch CLND im Vergleich zu LNE, was mit den Ergebnissen bei Patienten mit positivem Sentinel‐Lymphknoten vergleichbar ist. Allerdings war die lokale Kontrolle in der CLND‐Gruppe verbessert. Von allen 320 eingeschlossenen Patienten mit Lymphknoten‐Makrometastasen erhielten der Großteil der Patienten (n = 222; 69,4 %) eine CLND. Die große Heterogenität zwischen der CLND‐ und der LNE‐Gruppe ist auch auf den retrospektiven Charakter der Studie zurückzuführen. Dabei unterschieden sich insbesondere die Anzahl der histologisch bestätigten Lymphknotenmetastasen und die damit verbundenen Krankheitsstadien zwischen den beiden Gruppen. Da die Anzahl der Lymphknotenmetastasen das Überleben entscheidend beeinflusst,^18^, ^19^, ^21^, ^28^, ^29^ waren die Auswirkungen der Interventionen auf das Überleben teils innerhalb der gesamten Studienkohorte schwer zu differenzieren. Nach Anpassung für die Risikofaktoren mehr als drei positive Lymphknoten und der Dauer der adjuvanten Systemtherapie mittels multivariater Cox‐Proportional‐Hazards‐Regressionsanalyse zeigte die CLND ein im Vergleich zur LNE signifikant verlängertes RFS. Bezüglich der Art des Progresses wurden in der CLND‐Gruppe weniger regionale Lymphmetastasen‐Rezidive beobachtet, während Fernmetastasen, In‐transit‐Metastasen sowie Lokalrezidive im Bereich des Primärtumors in beiden Gruppen ähnlich verteilt waren. Diese Ergebnisse legen nahe, dass die Durchführung der CLND bei einem Teil der Patienten mit Lymphknoten‐Makrometastasen trotz der höheren Rate an postoperativen Komplikationen gerechtfertigt ist. Es ist davon auszugehen, dass dieser Effekt besonders bei den Patienten relevant ist, die keine adjuvante Strahlentherapie erhalten, welche ebenfalls das Risiko für ein Lokalrezidiv verringern könnte.^30^, ^31^, ^32^ Außerdem fiel auf, dass die adjuvante Strahlentherapie in den univariaten Cox‐Regressionsmodellen dieser Studie mit einem schlechteren RFS und OS verbunden war. Dies könnte darauf zurückzuführen sein, dass eine adjuvante Strahlentherapie insbesondere bei Vorliegen unabhängiger Risikofaktoren wie mehr als drei metastatische Lymphknoten, ein maximaler Lymphknotendurchmesser von mehr als 4 cm und einer Kapsel‐überschreitenden Lymphknotenmetastase empfohlen wird.^30^

Im Gegensatz zum RFS ergab die multivariate Cox‐Proportional‐Hazard‐Regressionsanalyse bezüglich des OS keinen Überlebensvorteil für die CLND gegenüber der LNE. Dies könnte auf die oben erwähnten lokalen Vorteile der CLND zurückzuführen sein. Andererseits beeinflussen weitere melanomspezifische Faktoren wie nachfolgende Therapien und auch nicht‐melanomspezifische Faktoren das OS erheblich, was das OS zu einem relativ unspezifischen Ergebnisparameter macht, der häufig durch das melanomspezifische Überleben ergänzt wird. Darüber hinaus waren die relativ kleine Studienpopulation und die recht kurze Nachbeobachtungszeit trotz möglicher klinischer Vorteile möglicherweise nicht ausreichend, um signifikante Auswirkungen auf das OS festzustellen.

Die Studie konnte zeigen, dass nach einem Rezidiv nach LNE nur bei einem kleinen Teil der Patienten eine Zweitlinien‐CLND durchgeführt wurde. Aus diesem Grund war es nicht möglich, den therapeutischen Nutzen einer Zweitlinien‐CLND zu bewerten.

Außerdem war die CLND im Vergleich zur LNE mit einer höheren Komplikationsrate assoziiert. Das Lymphödem war eine häufigere Komplikation nach CLND. Wie bereits in anderen Studien gezeigt wurde,^33^, ^34^ wurde bei inguinalen Eingriffen eine höhere Komplikationsrate als bei axillären oder zervikalen Eingriffen beobachtet. Aufgrund des größeren Anteils an inguinalen Eingriffen in der LNE‐Gruppe kann davon ausgegangen werden, dass die Gesamtkomplikationsrate für die LNE‐Gruppe im Vergleich zur CLND‐Gruppe möglicherweise überschätzt wurde und die Unterschiede in den Komplikationsraten nach Bereinigung um die Lokalisation der Eingriffe sogar noch größer sein könnten.

Besonders die unterschiedliche Verteilung der Lokalisationen der Eingriffe und die beobachtete Tendenz, eine CLND bei Patienten zu empfehlen, bei denen klinisch mehr als eine Lymphknoten‐Makrometastase vermutet wurden, stellen Limitationen dieser retrospektiven Analyse dar. Um einen umfassenden Einblick in die Rolle der CLND im Vergleich zur LNE bei Patienten mit konsekutiver adjuvanter Therapie zu gewinnen, wären deswegen prospektive klinische Studien erforderlich.

Diese Studie analysierte die Auswirkungen von CLND oder LNE bei Patienten mit Lymphknoten‐Makrometastasen, die eine adjuvante Systemtherapie erhielten. Mit der Zulassung neoadjuvanter Behandlungsschemata muss das chirurgische Vorgehen jedoch möglicherweise angepasst werden.^7^, ^8^, ^9^ Während in der NADINA‐ und SWOG 1801‐Studie Art und Umfang der Operation individuell gewählt werden konnten,^7^, ^8^ wurde in der PRADO‐Erweiterungskohorte der OpACIN‐neo‐Studie nur der Indexlymphknoten entfernt.^9^ Wie auch in dieser Studie beobachtet, führte der Verzicht auf die CLND in der PRADO‐Erweiterungskohorte der OpACIN‐neo‐Studie im Vergleich zur alleinigen Entfernung des Indexlymphknotens zu einer signifikant geringeren chirurgischen Morbidität.^9^ Die Ergebnisse unserer Studie, die einen RFS‐Vorteil der CLND gegenüber der LNE bei Melanompatienten unter adjuvanter Systemtherapie zeigten, unterstreichen, dass eine sorgfältige Bewertung des chirurgischen Verfahrens auch bei neoadjuvanten Behandlungsschemata erforderlich ist. Insbesondere bei Patienten ohne pathologisches Ansprechen, die etwa ein Viertel aller Patienten unter neoadjuvanter Behandlung ausmachen,^7^ kann die CLND immer noch eine wertvolle Behandlungsoption sein. Geeignete Biomarker, die das Ansprechen auf Immuntherapien vorhersagen, würden daher auch im Zeitalter der neoadjuvanten Behandlung dazu dienen, Patienten zu identifizieren, die von einer zusätzlichen CLND profitieren.

### Schlussfolgerungen

In dieser Studie wurde die CLND mit der selektiven Exzision klinisch verdächtiger Lymphknoten verglichen. Die CLND zeigte ein verbessertes NFS. Unter Berücksichtigung der Anzahl an positiven Lymphknoten und der Dauer der adjuvanten Systemtherapie traten nach CLND signifikant weniger Rezidive auf, während das OS nicht signifikant beeinflusst wurde. Gleichzeitig war die Zahl der Komplikationen in der CLND‐Gruppe signifikant höher.

Diese Ergebnisse sprechen für die Durchführung einer CLND vor adjuvanter Systemtherapie, mit dem Ziel, die lokale Tumorkontrolle zu verbessern. Aufgrund der höheren Rate an Komplikationen sollten jedoch individuelle Patientencharakteristika wie Alter und Allgemeinzustand in den Entscheidungsprozess miteinbezogen werden. Somit ist ein personalisierter Ansatz bei der Wahl des Umfangs des chirurgischen Eingriffs bei Patienten mit nodaler Makrometastasierung in der Ära adjuvanter Systemtherapien zu bevorzugen. Nach Zulassung neoadjuvanter Behandlungsschemata sollte dieses Vorgehen jedoch überdacht werden.

## DANKSAGUNG

Wir danken Dr. Victoria Kehl für ihre statistische Beratung bei der Analyse der Daten.

Open access Veröffentlichung ermöglicht und organisiert durch Projekt DEAL.

## INTERESSENKONFLIKT

K.E.M. erhielt Reiseunterstützung von Novartis und war Mitglied des Advisory Boards der Imnotech GmbH, die Patente an einem anti‐CD2‐ und einem anti‐CD7‐Single‐Domain‐Antikörper hält. J.C.H. erklärt Honorare von Amgen, Bristol Myers Squibb, Delcath, GlaxoSmithKline, Immunocore, Merck Sharp & Dohme, Novartis, Pierre Fabre, Sanofi und Sun Pharma außerhalb der eingereichten Arbeit. J.S. erklärt, dass keine Interessenkonflikte bestehen. M.E. erklärt Honorare und Reiseunterstützung von Bristol Myers Squibb, Immunocore, Novartis und Pierre Fabre außerhalb der eingereichten Arbeit. M.A.S. erklärt Honorare für Beratung und/oder Vortragstätigkeit und/oder Reiseunterstützung von Bristol Myers Squibb, Immunocore, Kyowa Kirin, Merck Sharp & Dohme, Novartis, Pierre Fabre, Sanofi, Regeneron, Sun Pharma und Takeda. J.O. erklärt, dass keine Interessenkonflikte bestehen. A.T. erhielt Forschungsmittel von Almirall Hermal sowie Berater‐, Referentenhonorare und/oder Reiseunterstützung von Almirall Hermal, Bristol Myers Squibb, Boehringer Ingelheim, GlaxoSmithKline, Janssen, Kyowa Kirin, Merck Sharp & Dohme, Pfizer, Pierre Fabre, Recordati Rare Diseases und Sanofi. D.T. berichtet über Berater‐, Referentenhonorare oder Reiseunterstützung von Bristol Myers Squibb, Roche, Novartis, Sanofi, Recordati, Kyowa Kirin, Sun Pharma und Pierre Fabre. J.K. erklärt, dass keine Interessenkonflikte bestehen. M.S. erhielt Beratungshonorare und Reiseunterstützung von Bristol Myers Squibb, Merck Sharp & Dohme, Kyowa Kirin, Novartis, Pierre Fabre, Sun Pharma und Immunocore. C.B. erklärt Honorare für Beratung und/oder Vortragstätigkeit von Almirall Hermal, Bristol Myers Squibb, Novartis, Merck Sharp & Dohme, Immunocore, Delcath, InflaRx, Pierre Fabre, Sanofi und Regeneron außerhalb der eingereichten Arbeit. C.P. erhielt Honorare und Reiseunterstützung von AbbVie, Bristol Myers Squibb, Pelpharma, Novartis, Leo Pharma, Galderma, Celgene, Iovance, Sun Pharma, Sanofi Genzyme, Merck Sharp & Dohme, Merck, Almirall, Pfizer, Pierre Fabre, DSD, AstraZeneca, Eli Lilly, Janssen, Takeda und Amazentis, die nicht mit dieser Arbeit in Zusammenhang stehen. T.B. erhielt Zuschüsse von Almirall, Celgene‐BMS, Lilly, Novartis, Sanofi Genzyme, Regeneron und Viatris; Beratungsgebühren von AbbVie, Alk‐Abelló, Almirall, Boehringer Ingelheim, Leo Pharma, Lilly, Novartis, Sanofi Genzyme und Viatris; Honorare von Alk‐Abelló, Almirall, GlaxoSmithKline, Leo Pharma, Lilly, Novartis, Sanofi Genzyme und Regeneron und ist Mitglied des Beirats von Alk‐Abelló, Almirall, Boehringer Ingelheim, Leo Pharma, Lilly, Novartis, Sanofi Genzyme und Viatris. O.D.P. erklärt Honorare und Reisekostenzuschüsse von Merck Sharp & Dohme, Kyowa Kirin, Pierre Fabre, Sanofi, Sun Pharma und Bristol Myers Squibb erhalten zu haben.

## Supporting information



Supplementary information
