# Supplementary material for: Komplette Lymphknotendissektion versus selektive Lymphknotenexstirpation bei Melanompatienten mit nodaler Makrometastasierung und adjuvanter Systemtherapie
Source: J Dtsch Dermatol Ges. 2026 Aug 4;24(8):1052–62. [Article in German] doi: 10.1111/ddg.15967_g (PMC13435065; doi:10.1111/ddg.15967_g)
Supplement: Supplementary file 1 [file DDG-24-1052-s001.docx]

| [[Online Supplement]]  TABLE S1 Langfristige Ergebnisse nach CLND vs. LNE. | | |
| --- | --- | --- |
|  |  |  |
|  | **CLND**  **(n = 222; 69,4 %)** | **LNE**  **(n = 98; 30,6 %)** |
| ***Progress*** |  | |
| Ja | 98 (44,1 %) | 51 (52,0 %) |
| Nein | 124 (55,9 %) | 47 (48,0 %) |
| ***Art von Progress*** |  |  |
| Regionale Lymphknotenmetastasierung | 16 (16,3 %) | 17 (33,3 %) |
| Fernmetastasierung | 74 (75,5 %) | 36 (70,6 %) |
| In-transit-Metastasierung oder Lokalrezidiv im Bereich des Primärtumors | 11 (11,2 %) | 7 (13,7 %) |
| **Tod** |  | |
| Ja | 42 (18,9 %) | 22 (22,4 %) |
| Nein | 180 (81,1 %) | 76 (77,6 %) |
| ***Rezidivfreies Überleben (RFS)*** |  |  |
| Median in Monaten | 43 | 30 |
| **Lymphknotenmetastasen-freies Überleben (NFS)** |  |  |
| Median in Monaten | Nicht erreicht | Nicht erreicht |
| ***Gesamtüberleben (OS)*** |  |  |
| Median in Monaten | Nicht erreicht | Nicht erreicht |

*Abk.:* CLND, komplette Lymphknotendissektion; LNE, Lymphknotenexstirpation

| TABELLE S2 Komplikationen nach CLND vs. LNE. | | | | | | | | |  | |  |
| --- | --- | --- | --- | --- | --- | --- | --- | --- | --- | --- | --- |
|  |  |  |  |  |  |  |  |  | |  | |
|  | **CLND (n = 222)** | | | | **LNE (n = 98)** | | | | | | |
| ***Komplikation nach CLND oder LNE*** | ***Gesamt (n = 222)*** | ***Inguinal (n = 64)*** | ***Axillär (n = 114)*** | ***Zervikal (n = 44)*** | ***Gesamt (n = 98)*** | ***Inguinal (n = 40)*** | ***Axillär (n = 31)*** | ***Zervikal (n = 19)*** | | ***Andere (n = 8)*** | |
| Ja | 84 (37,8 %) | 31 (48,4 %) | 42 (36,8 %) | 11 (25,0 %) | 20 (20,4 %) | 13 (32,5 %) | 5 (16,1 %) | 2 (10,5 %) | | 0 | |
| Nein | 129 (58,1 %) | 30 (46,9 %) | 69 (60,5 %) | 30 (68,2 %) | 72 (73,5 %) | 24 (60,0 %) | 25 (80,6 %) | 17 (89,5 %) | | 6 (75,0 %) | |
| Unbekannt | 9 (4,1 %) | 3 (4,7 %) | 3 (2,6 %) | 3 (6,8 %) | 6 (6,1 %) | 3 (7,5 %) | 1 (3,2 %) | 0 | | 2 (25,0 %) | |
| ***Art der Komplikation nach CLND oder LNE*** | ***Gesamt (n = 89)*** | ***Inguinal (n = 32)*** | ***Axillär (n = 46)*** | ***Zervikal (n = 11)*** | ***Gesamt (n = 20)*** | ***Inguinal (n = 13)*** | ***Axillär (n = 5)*** | ***Zervikal (n = 2)*** | | ***Andere (n = 0)*** | |
| Serom/Lymphozele | 35 (39,3 %) | 11 (34,4 %) | 21 (45,7 %) | 3 (27,3 %) | 12 (60,0 %) | 8 (61,5 %) | 4 (80,0 %) | 0 | | 0 | |
| Lymphatische Fistel | 5 (5,6 %) | 3 (9,4 %) | 2 (4,3 %) | 0 | 0 | 0 | 0 | 0 | | 0 | |
| Blutung/Hämatom | 7 (7,9 %) | 4 (12,5 %) | 2 (4,3 %) | 1 (9,1 %) | 2 (10,0 %) | 1 (7,7 %) | 0 | 1 (50,0 %) | | 0 | |
| Lymphödem | 38 (42,7 %) | 14 (43,8 %) | 20 (43,5 %) | 4 (36,4 %) | 4 (20,0 %) | 3 (23,1 %) | 1 (20,0 %) | 0 | | 0 | |
| Fazialisparese | 3 (3,3 %) | – | – | 3 (27,3 %) | 1 (5,0 %) | – | – | 1 (50,0 %) | | 0 | |
| Infektion | 1 (1,1 %) | 0 | 1 (2,2 %) | 0 | 1 (5,0 %) | 1 (7,7 %) | 0 | 0 | | 0 | |

*Abk.:* CLND, komplette Lymphknotendissektion; LNE, Lymphknotenexstirpation

[Translation Fig 1+2]

Fig 1:

survival probalility

Überlebenswahrscheinlichkeit

Time [months]

Zeit [Monate]

Fig 2:

% of patients

% Patienten

complication	Komplikation

no complication	keine Komplikation

\# of complications/100 patients

\# Komplikationen/100 Patienten

Serom

Lymphödem

Fistel

Hämatom

Nervenlähmung

Infektion
